# The various roles of TREM2 in cardiovascular disease

**DOI:** 10.3389/fimmu.2025.1462508

**Published:** 2025-02-27

**Authors:** Shuai Wang, Chenghui Cao, Daoquan Peng

**Affiliations:** Second Xiangya Hospital of Central South University, Cardiovascular Medicine, Changsha, China

**Keywords:** macrophage, TREM2, atherosclerosis, myocardial infarction, sepsis-induced cardiomyopathy, HFpEF

## Abstract

Triggering receptor expressed on myeloid cells-2 (TREM2) is a transmembrane immune receptor that is expressed mainly on macrophages. As a pathology-induced immune signaling hub, TREM2 senses tissue damage and activates immune remodeling in response. Previous studies have predominantly focused on the TREM2 signaling pathway in Alzheimer’s disease, metabolic syndrome, and cancer. Recent research has indicated that TREM2 signaling is also activated in various cardiovascular diseases. In this review, we summarize the current understanding and the unanswered questions regarding the role of TREM2 signaling in mediating the metabolism and function of macrophages in atherosclerosis and various models of heart failure. In the context of atherosclerosis, TREM2 signaling promotes foam cell formation and is crucial for maintaining macrophage survival and plaque stability through efferocytosis and cholesterol efflux. Recent studies on myocardial infarction, sepsis-induced cardiomyopathy, and hypertensive heart failure also implicated the protective role of TREM2 signaling in cardiac macrophages through efferocytosis and paracrine functions. Additionally, we discuss the clinical significance of elevated soluble TREM2 (sTREM2) in cardiovascular disease and propose potential therapies targeting TREM2. The overall aim of this review is to highlight the various roles of TREM2 in cardiovascular diseases and to provide a framework for therapeutic strategies targeting TREM2.

## 1 Introduction

Macrophages represent one of the most numerous and diverse leukocyte types in the body (1). They are important regulators of many cardiovascular diseases. Macrophages not only trigger damaging inflammatory responses but also mediate tissue repair and cardiac regeneration (2). Recent molecular techniques, such as single-cell mass cytometry by time-of-flight, have revealed significant complexity within macrophage populations (3). Distinct macrophage populations may mediate inflammatory or reparative macrophage behaviors (4). A deeper understanding of the diverse functions of cardiac macrophages will be essential for the development of targeted therapies to mitigate injury and orchestrate recovery.

Recently, via single-cell RNA sequencing, a subpopulation of nonproinflammatory foamy macrophages highly expressing triggering receptor expressed on myeloid cells-2 (*TREM2*) was identified in atherosclerotic plaques, but its importance remains to be elucidated (5, 6). Interestingly, single-cell RNA sequencing of cardiac immune cells also revealed upregulated *Trem2* expression in subpopulations of macrophages in animal models of myocardial infarction (MI), hypertension-induced heart failure with preserved function (HFpEF), and septic cardiomyopathy (7–9). *Trem2^+^* macrophages were found to promote recovery from cardiac injury and improve cardiac function in the above disease models (8–11).

TREM2 is a transmembrane receptor belonging to the immunoglobin superfamily. As a hub of myeloid cell immune activity, TREM2 signaling helps to maintain normal myeloid cell differentiation and survival under physiological conditions, whereas under conditions of acute or chronic injury, TREM2 regulates the phenotype and function of myeloid cells in response to injury (12, 13). In different scenarios, TREM2 signaling mediates various physiological processes, including phagocytosis, resistance to proinflammatory stimuli, the promotion of myeloid cell survival under conditions of stress, and phenotypic transformation (14).

Prior studies have mainly concentrated on the function and regulation of TREM2 in the development of neurodegenerative disorders, particularly Alzheimer’s disease (14). Nonetheless, the involvement of TREM2 in cardiovascular disease has not been thoroughly explored. A recent review has highlighted TREM2’s protective role in the heterogenous population of macrophages during inflammation following myocardial infarction (15). However, there is conflicting evidence regarding the importance of the TREM2 signaling pathway in macrophages related to atherosclerosis. Additionally, the implications of TREM2 signaling in macrophages during cardiac injury responses across various heart failure models still require further investigation. This review aims to compile existing knowledge and identify gaps regarding the Trem2 signaling pathway in both atherosclerosis and cardiomyopathy, while also considering the potential for therapeutic modulation of TREM2 signaling in cardiovascular disease.

## 2 TREM2 macrophages in atherosclerosis

### 2.1 Macrophages and plaque stability

Plaque stability is a key determinant of the prognosis of atherosclerotic cardiovascular diseases (16). Currently, therapeutic approaches for atherosclerotic diseases focus primarily on controlling risk factors (such as intensive lipid lowering). Although the combination of statins and proprotein convertase subtilisin/kexin (PCSK9) inhibitors can significantly reduce low-density lipoprotein cholesterol (LDL-C), plaque regression is limited (16). Thus, patients with atherosclerotic cardiovascular disease (ASCVD) still face a high residual risk (17). Macrophage-mediated plaque inflammation is recognized as an important factor influencing plaque progression and regression (18–21).

It was previously thought that macrophages within plaques possess both anti-inflammatory and proinflammatory phenotypes and play different roles in the progression and regression of plaques. M1 macrophages, which are activated *in vitro* by stimulation of Toll-like receptor ligands, lipopolysaccharide (LPS) and interferon gamma (INF-γ). M1 macrophages express the proinflammatory transcription factors NF-κB and STAT-1; secrete the proinflammatory cytokines IL-1β, IL-6, and TNF-α; and promote plaque progression. Alternatively, macrophages activated *in vitro* by IL-4 and IL-13 known as M2 macrophages. M2 macrophages highly express CD163, mannose receptor 1, resestin like-β, and arginase-1; secrete anti-inflammatory factors such as the IL-1 receptor antagonist IL-10 and collagen; and promote plaque regression (4). The traditional dichotomy of macrophage phenotypes is overly simplistic. With the application of mass cytometry and single-cell RNA sequencing technologies, more complex subgroups of macrophages have been discovered in atherosclerotic plaques (5, 6, 22).

### 2.2 TREM2^hi^ macrophages in atherosclerotic plaques

**Figure 1:**
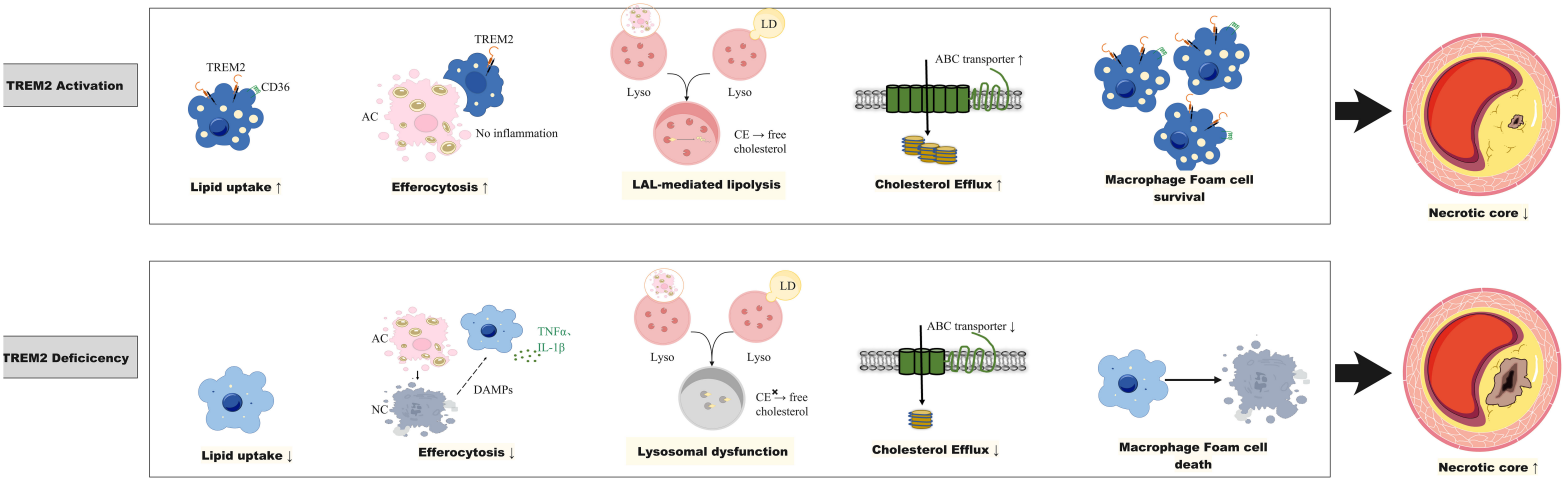
The role of Trem2 in atherosclerosis. TREM2 in plaque macrophages is involved in lipid uptake and foam cell formation, the efferocytosis of apoptotic or necrotic cells to limit plaque inflammation, maintaining lysosomal cholesterol homeostasis to prevent cholesterol ester (CE) accumulation and promoting cholesterol efflux through the upregulation of ATP-binding cassette A1 (ABCA1)/ABCG1. TREM2 activation promotes foam cell survival and reduces necrotic core, whereas TREM2 deficiency leads to foam cell death and an increase in the necrotic core. ABC transporter, ATP binding cassette transporter; CE, cholesterol ester; DAMPs, damage-associated molecular patterns; LAL, lysosomal acid lipase; LD, Lipid droplet; IL-1β, interleukin-1β; Lyso, lysosome; TNF-α, tumor necrosis factor alpha; TREM2, triggering receptor expressed on myeloid cells-2.

Recently, a subset of macrophages TREM2^hi^, which are characterized by markers of *Trem2, Cd9, Ctsd*, and *Spp1*, was identified in human and mouse atherosclerotic plaques (5, 6, 23–25). TREM2, which acts as a lipid-sensing receptor, can bind to lipoprotein ligands such as apolipoprotein (ApoE) or phospholipids (26, 27). After TREM2 binds to lipid ligands, the p38/mitogen-activated protein kinase (MAPK)-proliferator-activated receptor γ (PPARγ) signaling pathway is activated, and CD36 expression is upregulated, which is a receptor that mediates cholesterol uptake (28). Previous studies have shown that TREM2^hi^ macrophages are lipid-rich foam cells with a low inflammatory state (5, 6, 29). A recent study employing trajectory analysis, ligand-receptor interaction analysis, and functional experiments has confirmed that Trem2^hi^ macrophage can transform into pro-inflammatory lipid-laden macrophages (PLIN2^hi^/TREM1^hi^), which are implicated in the progression of atherosclerotic lesions (30).The overexpression of TREM2 in cultured vascular smooth muscle cells (SMCs) and macrophages promote lipid uptake, exacerbates lipid influx and foam cell formation (28, 31). In an atherosclerotic mouse model, the density of Trem2-positive foam cells in aortic plaques increased in a time-dependent manner after *ApoE* knockout (*ApoE* ^-/-^) mice were fed a high-fat diet (HFD) (28). Moreover, compared with *ApoE* ^-/-^ mice, *Trem2* ^-/-^/*ApoE* ^-/-^ double-knockout mice presented significantly reduced atherosclerotic lesion sizes, foam cell numbers, and lipid burdens after HFD feeding (28). Therefore, TREM2 is speculated to exacerbate atherosclerosis development. However, the role of TREM2^hi^ macrophages in atherosclerosis is controversial since other evidence suggests that TREM2^hi^ macrophages may play a role in stabilizing plaques and promoting plaque regression (**Figure 1**).

### 2.3 Activating TREM2 signaling promotes cholesterol efflux from macrophages

The process by which cholesterol in macrophages is transported to high-density lipoprotein (HDL) through the cholesterol transporters ATP binding Cassette A1 and G1 (ABCA1/ABCG1) is known as cholesterol efflux. Promoting cholesterol efflux from macrophages decreases cholesterol accumulation in foam cells, suppresses inflammation and promotes plaque stability and plaque regression (32, 33). Research on chronic demyelinating neurological diseases, ischemic stroke and neurotoxicity has shown that TREM2 promotes cholesterol efflux in microglia (27, 34–36). Single-cell transcriptomic and chromatin accessibility analysis techniques revealed that in human aortic plaques, TREM2^hi^ macrophages highly express the cholesterol efflux transporters *ABCA1* and *ABCG1* (5). In addition, TREM2-dependent *Abca1* and *Abcg1* expression and cholesterol efflux have been reported to explain the sexually dimorphic effect of serum amyloid A3 (SAA3) on atherosclerosis (37). SAA3 is predominantly an extrahepatically expressed acute-phase protein in response to acute and chronic inflammatory stimuli (38, 39). A previous report suggested that SAA3 is proatherogenic in male *ApoE* ^-/-^ mice, as evidenced by worsened atherosclerosis when *Saa3* was overexpressed and by improved atherosclerosis when *Saa3* was silenced (40). However, these effects have been reported only in male mice but not in female mice (40). Chait et al. (37) showed that *Saa3* deficiency differentially altered *Trem2* expression in macrophages from male and female mice. Although *Saa3* deficiency in male *Ldlr^-/-^* mice reduced aortic and aortic sinus atherosclerosis and increased *Trem2* transcript levels in bone marrow-derived macrophages (BMDMs), *Saa3^-/-^* knockout in female *Ldlr^-/-^* mice increased aortic atherosclerosis and decreased *Trem2* expression in BMDMs. In addition, BMDMs from male *Saa3* ^-/-^ mice presented increased expression of the cholesterol transporters *Abca1* and *Abcg1*, as well as increased cholesterol efflux to HDL. In contrast, *Saa3* deficiency in female BMDMs had no effect on cholesterol transporter gene expression or cholesterol efflux. The results of the study by Chait et al. support the notion that TREM2 in macrophages promotes cholesterol efflux and plays a role in combating atherosclerosis (37).

### 2.4 TREM2 is involved in maintaining plaque macrophage efferocytosis

Efferocytosis is a process through which diseased and dying cells are engulfed by phagocytes for clearance (41). In healthy arteries, cells undergo apoptosis as a result of lipid ingestion after foam cell formation or due to homeostatic cell turnover (41). Macrophage efferocytosis is impaired in atherosclerotic cardiovascular disease (42). Efferocytosis failure accelerates atherogenesis by permitting the accumulation of debris and apoptotic cells within the arterial subintima and media (42, 43). The resulting plaque growth not only increases the degree of stenosis of the affected vessel but also increases plaque vulnerability to rupture given that the accumulation of this inflammatory tissue occurs within the necrotic core (41, 44, 45). Recent studies have shown that macrophage efferocytosis is dependent on TREM2 and that the absence or downregulation of TREM2 results in defects in tissue macrophage efferocytosis (46, 47). In addition, a recent study demonstrated that hematopoietic or global deficiency of TREM2 increased necrotic core formation in early atherosclerosis, while TREM2 agonism decreased it (48). In atherosclerosis-prone mice, treatment with Trem2 agonism AL002a promote macrophage survival and improves feature of plaque stability (49).These findings underscore the essential role of TREM2 in maintaining the balance between foam cell death and the clearance of dead cells in atherosclerotic lesions (48, 49). Further mechanistic studies on how TREM2 regulates efferocytosis revealed that TREM2 can bind to phosphatidylserine and phosphatidylcholine, both of which are “eat me” signaling molecules that are released by apoptotic cells, suggesting that TREM2 itself may be a scavenger receptor responsible for recognizing and clearing apoptotic cells (50). In addition, the TREM2-mediated signaling pathway can upregulate the expression of macrophage “recognition” receptors for apoptotic cells, such as CD36 (28, 51). Therefore, the observed TREM2^hi^ macrophages in mouse models and human atherosclerotic lesions may reflect the active efferocytotic function of plaque macrophages.

### 2.5 TREM2 regulates macrophage lysosomal cholesterol metabolism

Lysosomes are at the center of the cholesterol homeostasis regulatory network (52). When macrophages take up modified lipoproteins or ingest apoptotic cells, they face a substantial cholesterol load. Excessive cholesterol must be processed within lysosomes to avoid cytotoxic cholesterol accumulation, which may cause oxidative stress, mitochondrial dysfunction and inflammasome activation. Intracellular cholesterol needs to be re-esterified for storage or exported via the cholesterol efflux pathway. In lysosomes, cholesteryl ester is hydrolyzed into free cholesterol by lysosomal acid lipase (LAL) and is then distributed among various organelles via the Niemann−Pick C (NPC) 1 and NPC2 proteins (52). Lysosomal cholesterol hydrolysis, which is mediated by LAL, leads to the timely production of oxysterols, which are endogenous liver X receptor (LXR) ligands. LXR, a nuclear receptor, plays pivotal roles in the transcriptional regulation of lipid metabolism and efferocytosis (53–55). A deficiency in LAL impaired the production of LXR ligands, resulting in reduced expression of the cholesterol transporters *ABCA1* and *ABCG1* and the efferocytosis recognition receptor *MerTK*, thereby impeding subsequent macrophage cholesterol efflux and efferocytosis (56).

TREM2 was recently reported to play an important role in maintaining normal lysosomal function and cholesterol metabolism homeostasis (57, 58). TREM2 loss-of-function mutations lead to significant lysosomal dysfunction and downregulated expression of genes involved in lipid metabolism in induced pluripotent stem cell (iPSC)-derived microglia from patients with Nasu-Hakola disease (NHD) (57). In animal studies, knocking out *Trem2* in mice led to reduced expression of microglial lysosomal function-related genes and the accumulation of cholesterol esters (58). These findings suggest TREM2-dependent expression of genes involved in lysosomal cholesteryl ester hydrolysis and cholesterol export. Although this has not been confirmed in the context of atherosclerosis, the high expression of TREM2 on foam cells is hypothesized to be an adaptive response to cholesterol load, through which normal lysosomal cholesterol processing is maintained to prevent toxic cholesterol accumulation.

In summary, the significance of TREM2^hi^ macrophages in atherosclerosis is still under debate since opposite evidence exists. Given that foam cell formation reflects the scavenging role of macrophages to clear subintimal lipid accumulation and that TREM2 is important for maintaining macrophage cholesterol efflux, efferocytosis, and lysosomal cholesterol metabolism, it is speculated that high expression of TREM2 is protective in atherosclerosis. However, further studies in different animal models of atherosclerosis are needed.

## 3 TREM2^hi^ macrophages in myocardial repair and heart failure

### 3.1 Efferocytosis and metabolic remodeling of TRME2^hi^ macrophages preserve myocardial function after MI

Macrophages play a central role in the response to injury after MI. Post MI, macrophages follow a biphasic response to injury, transiting from a proinflammatory phenotype toward a reparative phenotype, which has been classically defined as M1-to-M2 polarization (59). However, macrophages in the post-MI heart exhibit heterogeneous subtypes of surface markers and functions that lie outside the M1/M2 spectrum (59, 60).

Recent studies utilizing spatial transcriptomics and single-cell RNA sequencing in a mouse model of MI identified a group of macrophages with high *Trem2* expression (Trem2^hi^ macrophages). These cells highly express anti-inflammatory genes, cytokines, and chemokines such as *Il10, Tgfb1, Cx3cr1, Alox15*, and *Arg1* (7). Trem2^hi^ macrophages that appear in the myocardium 3–7 days post-MI are thought to be recruited from the circulation because the expression of *Trem2* in circulating monocytes increases synchronously (10, 11). In addition, the use of a CCR2 chemokine antagonist inhibits the migration of TREM2^hi^-BMDMs into the heart (11).

A key function of Trem2^hi^ macrophages in the infarcted myocardium is efferocytosis. It is well known that efferocytosis after MI is an essential aspect of healing following cardiac injury and that it stabilizes the myocardial wall to prevent wall rupture (2, 61, 62). Specifically, knocking out *Trem2* in mice significantly suppressed the efferocytosis of dying cells, aggravated cardiac dysfunction and increased mortality, whereas the overexpression of *Trem2* or local myocardial injection of an adenoviral vector expressing *Trem2* restored the clearance of tissue debris and cardiac function (10, 11).

TREM2 also plays a critical role in mediating efferocytosis-dependent intracellular signaling. As a transmembrane receptor, TREM2 mediates intracellular signaling by associating with the adaptor proteins DAP12 and DAP10 to recruit the intracellular signaling molecules spleen tyrosine kinase (Syk) and phosphoinositide 3-kinase (PI3K) for signal transduction (63). Gong et al. (11) recently showed in an MI mouse model accompanied by efferocytosis that the TREM2-SYK-SMAD signaling pathway was activated, resulting in remodeling of cardiac macrophage metabolism and the secretion of protective cytokines. Activation of TREM2-SYK-SMAD signaling suppressed the transcription of the mitochondrial inner membrane NAD^+^ transporter solute carrier 25 member 53 (SLC25A53), thus impeding the entry of NAD^+^ into mitochondria, which caused reduced generation of α-ketoglutarate from isocitrate via the tricarboxylic acid (TCA) cycle. The consequence of this metabolic remodeling is the accumulation of the bypass derivative itaconate in the cytosol, which is secreted into the microenvironment and acts on adjacent cardiomyocytes and fibroblasts (11). Itaconate has been demonstrated to exert a protective effect on myocardial infarction by inhibiting the apoptosis of cardiomyocytes in an ischemic environment, promoting the proliferation of fibroblasts, and suppressing inflammation (11, 64–67).

Overall, these studies suggest that TREM2^hi^ macrophages play a protective role in the response to myocardial infarction by promoting healing and tissue remodeling through efferocytosis of apoptotic and necrotic cardiomyocytes and secretion of anti-inflammatory factors that promote fibrocyte proliferation.

### 3.2 TREM2^hi^ in cardiac macrophages contribute to the recovery of cardiac function during sepsis-induced cardiomyopathy through the scavenging of defective mitochondria

Sepsis can lead to cardiac pump dysfunction, commonly referred to as sepsis-induced cardiomyopathy (SICM). Although there is no consensus on the definition of SICM, a recent systematic review and meta-analysis comprising 16 studies defined SICM as new-onset left ventricular (LV) systolic dysfunction occurring with the onset of sepsis, with a pooled prevalence of 20% (68). The mechanism underlying sepsis-induced myocardial injury involves cardiac mitochondrial dysfunction (69). A recent study has demonstrated that cardiomyocytes can expel damaged mitochondria in the form of vesicles, known as exophers, into the extracellular space of the heart (70). Given that free mitochondria and mitochondrial DNA can induce cardiac damage, the elimination of exophers containing cardiomyocyte-derived mitochondria, a process termed heterophage, is crucial for maintaining cardiac homeostasis and restoring optimal cardiac function (70, 71).

In a cecal ligation and puncture (CLP) sepsis mouse model, single-cell RNA sequencing with fate mapping techniques identified a subgroup of cardiac-resident macrophages highly expressing Trem2 (Trem2^hi^-CD163+RETNLA+ macrophages). This group of Trem2^hi^ macrophages highly expresses a transcriptome related to phagocytosis according to Gene Ontology (GO) enrichment analysis. Confocal and three-dimensional (3D) images revealed that TREM2^hi^ macrophages engulfed exophers containing cardiomyocyte-derived mitochondria in septic hearts. In addition, TREM2^hi^ macrophages robustly eliminate cardiomyocyte-derived dysfunctional mitochondria through lysosomes (9).

To determine the physiological role of TREM2^hi^ macrophages in SICM, CLP was performed in Trem2 knockout (*Trem2* ^-/-^) mice and wild-type littermates as controls. Cardiac macrophages from *Trem2* ^-/-^mice had reduced phagocytotic gene signatures, as evidenced by scRNA-seq data analysis. In addition, *Trem2* ^-/-^ septic mice exhibited impaired ability to clear damaged mitochondria. In addition, compared with WT controls, *Trem2* ^-/-^ septic mice had notably increased mortality and worse cardiac function (9). These results suggest that TREM2 is essential for macrophages to protect cardiac function after sepsis. Interestingly, the intraperitoneal injection of *Trem2* ^hi^ macrophages into *Trem2* ^-/-^ septic mice significantly enhanced cardiac function and improved cardiac injury and inflammation (9).

Taken together, these results suggest that TREM2^hi^ cardiac macrophages contribute to the recovery of cardiac function during SICM through the scavenging of defective mitochondria. The administration of TREM2^hi^ macrophages or the induction of TREM2 expression in macrophages may be a potential therapeutic approach for preventing and rescuing cardiac dysfunction in sepsis patients.

### 3.3 Protective role of TREM2 in hypertension induced HFpEF

Increasing evidence suggests that HFpEF is associated with a proinflammatory state (72, 73). Previous studies have suggested that cardiac macrophages contribute to diastolic dysfunction through the secretion of profibrotic cytokines in an HFpEF mouse model and that blockade of macrophage recruitment prevents diastolic dysfunction in a pressure overload mode (74–77). However, embryonic-derived cardiac-resident macrophages are important for initiating adaptive responses and preserving cardiac function during hypertensive stress (78). Therefore, a deeper understanding of the diverse functions of cardiac macrophages will be essential for the development of targeted therapies for HFpEF.

**Figure 2:**
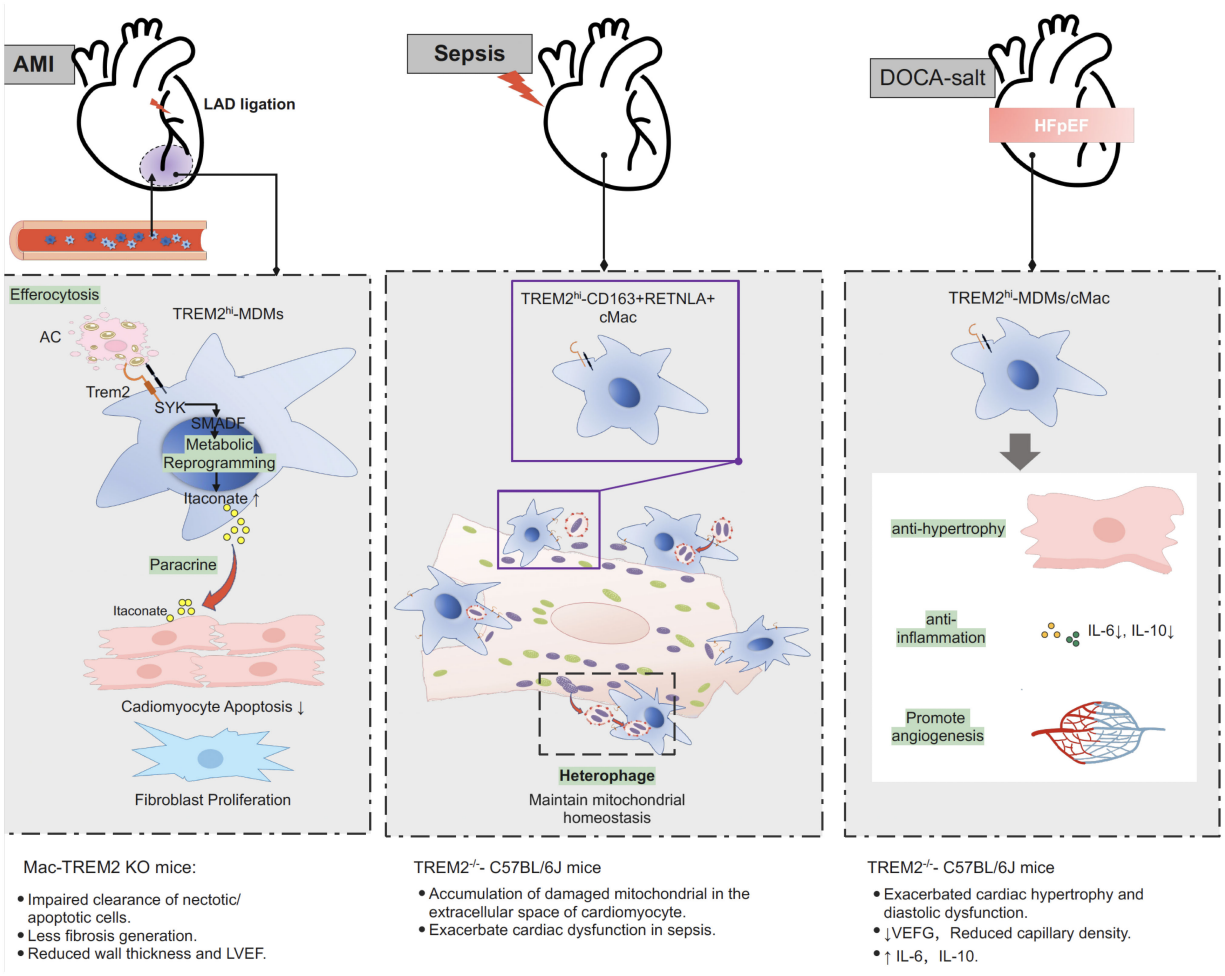
Protective role of Trem2 in different models of myocardial injury. After myocardial infarction, Trem2^hi^ macrophage is responsible for efferocytosis of apoptotic and necrotic cardiomyocytes. Efferocytosis-dependent activation of TREM2-SYK-SMAD signaling pathway remold the metabolism of macrophage and promote section of itaconate, inhibiting the apoptosis of cardiomyocytes in an ischemic environment, promoting the proliferation of fibroblasts, and suppressing inflammation. Specific knocking out *Trem2* in macrophage (LysM^Cre^TREM^flox/flox^, also termed Mac-TREM2 KO) resulted in impaired clearance of necrotic/apoptotic cell after MI, less fibrosis generation, reduced wall thickness and LVEF. In sepsis-induced cardiomyopathy, TREM2^hi^ macrophage contribute to the maintaining of mitochondrial homeostasis and recovery of cardiac function through the scavenging of defective mitochondria. Mice with complete knockout of the TREM2 gene (TREM2^-/–^C57BL/6J) resulted in accumulation of damaged mitochondria in the extracellular space of cardiomyocyte and exacerbated cardiac function in sepsis. In animal model of DOCA-salt induced hypertensive HFpEF, TREM2 expression was significantly upregulated in macrophage. Deletion of Trem2 (TREM2^-/–^C57BL/6J) lead to exacerbated cardiac hypertrophy and diastolic dysfunction, reduced capillary density, reduced expression of proangiogenic genes and increased expression of proinflammatory cytokines in macrophage. AMI, acute myocardial infarction; DOCA, deoxycorticosterone acetate; HFpEF, heart failure with preserved ejection fraction; LAD, left anterior descending coronary artery; MDMs, monocyte derived macrophages; cMac, cardiac macrophage; LVEF, left ventricular ejection fraction; PI3K, phosphoinositide 3-kinase; SYK, signaling molecules spleen tyrosine kinase; Trem2, triggering receptor expressed on myeloid cells-2.

Recently, in a mouse model of HFpEF driven by hypertension, Trem2-expressing macrophages were found to be protective in cardiac remodeling (8). The mouse model of hypertension induced by deoxycorticosterone acetate (DOCA) salt displayed clinical features of HFpEF, including diastolic dysfunction, reduced exercise tolerance, and pulmonary congestion (8). Cellular indexing of transcriptomes and epitopes by sequencing (CITE-Seq) revealed that DOCA-salt-treated mice exhibited significant upregulation of *Trem2* gene expression in cardiac macrophages (8). To determine the role of Trem2 in HFpEF, *Trem2^+/+^* and *Trem2^-/-^* mice were subjected to DOCA-salt treatment. Compared with wild-type control mice, mice with genetic deletion of the *Trem2* gene exhibited more severe cardiac hypertrophy and diastolic dysfunction, and no evidence of wall thinning or dilation was observed. Trem2-deficient mice also exhibit exacerbated cardiac hypertrophy and a decrease in cardiac capillary density (8). In addition, *Trem2*-deficient macrophages exhibit reduced expression of proangiogenic genes and increased expression of proinflammatory cytokines (8). These results suggest a protective role of Trem2 in hypertension-induced HFpEF (**Figure 2**).

## 4 Soluble TREM2

The extracellular domain of TREM2 is cleaved at H157-S158 by the proteases a-secretases disintegrin and metalloproteinase domain-containing protein 10 (ADAM10) and ADAM17, which results in the production of soluble TREM2 (sTREM2) (14). The remaining C-terminal fragment on the cell membrane is removed via γ-secretase processing. Moreover, sTREM2 can also be derived from the translation of another transcript lacking a transmembrane domain (79).

Currently, the binding of TREM2 to its ligand is believed to trigger the cleavage of sTREM2 and the removal of TREM2 from the cell membrane. Cells need to continuously mature and transport newly synthesized TREM2 proteins to the cell surface to maintain sustained receptor activity. The levels of sTREM2 in cerebrospinal fluid and plasma may reflect the contact, activation, and cleavage of the receptor TREM2 with its ligand, as well as the flow of new TREM2 proteins through the transport pathway (80).

Therefore, an increase in sTREM2 levels may reflect the extent of the macrophage response to injury and may serve as a biomarker for disease diagnosis and prognosis. For example, in nonalcoholic fatty liver disease and neuroinflammatory diseases, an increase in sTREM2 levels in blood or cerebrospinal fluid is correlated with disease severity. Therefore, sTREM2 may be a potential biochemical marker for disease diagnosis and severity assessment (81–84). In cardiovascular diseases, serum sTREM2 levels are significantly greater in patients with coronary heart disease than in healthy controls (85), and an increase in serum sTREM2 is associated with an increased risk of cardiovascular death in patients with coronary heart disease; thus, sTREM2 could serve as a marker for predicting plaque rupture (86). In patients with HFpEF, serum sTREM2 levels are significantly greater than those in patients without heart failure, and sTREM2 levels are independently associated with heart failure status (8).

Can cleaved sTREM2 bind to other cell surface transmembrane ligands to perform its biological functions? Recent research on Alzheimer’s disease suggested that sTREM2 can bind to microglia and promote their proliferation, migration, and aggregation around plaques, thus playing a neuroprotective role (87, 88). *In vitro* studies have shown that sTREM2 can affect the expression of inflammatory factors in THP-1-derived macrophages but has different effects on macrophages of different phenotypes: sTREM2 promotes the expression of proinflammatory factors in steady-state M0 and anti-inflammatory phenotype M2 macrophages while inhibiting the expression of inflammatory factors in proinflammatory phenotype (M1) macrophages (89). These findings suggest that sTREM2 may activate the macrophage response to damage, thereby limiting excessive inflammatory damage. In an MI mouse model, sTrem2 levels began to increase on the third day after myocardial infarction, reaching a peak level on the seventh day (7). *In vitro* experiments revealed that sTrem2 affects the phenotype of thioglycolate-elicited peritoneal macrophages. sTrem2 treatment reduces the expression of the proinflammatory genes *Il1b* and *Nos2* and increases *Arg1* expression (7). An *in vivo* study revealed that the injection of sTrem2 into the peri-infarct area of MI model mice significantly improved heart function and ventricular remodeling (7).

## 5 The potential of TREM2 as a therapeutic target for cardiovascular disease

In the context of CVD and HF, macrophages play active roles in the healing process following a wide range of injuries, ranging from inflammation to fibrosis and tissue remodeling. As the hub of pathological immune signaling, TREM2 is an attractive therapeutic target for cardiovascular disease (90). Recent preclinical studies have shown that immediate intramyocardial injection of recombinant adenovirus encoding full-length mouse TREM2 or sTREM2 significantly improves cardiac function in mice with myocardial infarction following ligation of the left anterior descending (LAD) coronary artery (7, 10, 11). Additionally, the injection of TREM2^hi^Mac1 cells into the pericardial cavity following CLP significantly alleviated cardiac dysfunction in mice with sepsis (9).

Recent studies have demonstrated that activated microglia regulate the progression of diseases such as hypertension, myocardial infarction, and ischemia/reperfusion injury by modulating autonomic nervous system activity following neuroinflammation (91). The overexpression of microglial TREM2 mitigates the inflammatory response of microglia and prevents the continuous activation of the sympathetic nervous system, a primary contributor to pathological cardiac remodeling. This suggests that targeting microglial TREM2 may represent a promising therapeutic approach for cardiovascular diseases (92).

Future strategies targeting TREM2 include the following: 1. Directly targeting the receptor’s active domain via specific monoclonal antibodies or small molecules to activate downstream signaling. A project led by Alector, LLC, using an agonistic monoclonal antibody against TREM2 (AL002) has completed phase I clinical trials (93). 2. The targeting conditions and/or tissue-specific TREM2 ligands can be used to direct therapeutics to the precise location at which treatment is needed. However, further understanding of the specific ligands of TREM2 in different tissues is still needed. 3. As TREM2 is continuously cleaved by ADAMs, inhibiting this cleavage is another potential target of treatment. A research team from Germany targeted the cleavage site of TREM2, thereby interfering with ADAM receptor cleavage, increasing the concentration of the receptor on the membrane, and enhancing its signaling activity. The team developed an antibody, 4D9, that binds to the stalk region of TREM2, reduces its proteolytic cleavage, and activates TREM2 signaling (94).

## 6 Summary

In summary, this review summarizes recent studies on the significance of TREM2 signals in cardiovascular disease, including 1) the impact of the TREM2 cellular pathway on macrophage metabolism and function, as well as the conflicting evidence concerning its role in atherosclerosis; 2) the latest research progress on the importance of macrophages with high TREM2 expression in the recovery of myocardial injury in different models of heart failure; 3) soluble TREM2 (sTREM2) as a potential prognostic biomarker in cardiovascular diseases; and 4) the prospects and challenges of potential treatments targeting TREM2.

The challenge for future interventions targeting TREM2 lies in how to specifically upregulate TREM2 expression in cardiac or plaque macrophages while avoiding potential side effects in other tissues, such as the harmful anti-inflammatory and immunosuppressive activities of TREM2, which promote tumor growth and immune escape (95–97).
